# The efficacy of a nutritional supplement containing green‐lipped mussel, curcumin and blackcurrant leaf extract in dogs and cats with osteoarthritis

**DOI:** 10.1002/vms3.779

**Published:** 2022-03-10

**Authors:** Ronald Jan Corbee

**Affiliations:** ^1^ Department of Clinical Sciences Faculty of Veterinary Medicine Utrecht University Utrecht The Netherlands

**Keywords:** canine, feline, greenshell mussel, osteoarthrosis, Synopet

## Abstract

**Background:**

Osteoarthritis is a common disease in dogs and cats, and the search for novel treatment options is needed. The combination of green‐lipped mussel, curcumin and blackcurrant leaf extract has to date not been studied in dogs and cats.

**Objectives:**

The aim of this study was to test the effect of a supplement containing green‐lipped mussel (*Perna canaliculus*), curcumin (*Curcuma longa*) and blackcurrant (*Ribes nigrum*) leaf extract on locomotion and behaviour in client‐owned dogs and cats suffering from mild to moderate osteoarthritis.

**Methods:**

To this end, 32 dogs and 16 cats were enrolled in a double‐blinded, randomised, crossover, placebo‐controlled trial for 10 weeks in cats and 16 weeks in dogs. Outcome parameters were the Helsinki Chronic Pain Index (HCPI) by pet owners in dogs and cats, Canine OsteoArthritis Staging Tool (COAST) by a veterinarian in dogs and Force Plate Analysis (FPA) in 18 dogs.

**Results:**

In dogs, the COAST improved significantly in the supplement group compared to baseline but was not different than the placebo group. In cats, the ability to groom, activity level, playfulness and walking up the stairs improved in the supplement group. No differences were found on HCPI scores and FPA in dogs. Several non‐responders were noted in both species, which were irrespective of the stage of osteoarthritis.

**Conclusions:**

Overall, the supplement had only partial positive effects in client‐owned dogs and cats with mild to moderate osteoarthritis. Further research with a larger sample size and longer duration is needed to expand these findings.

## Graphical abstract

The aim of this study was to test the effect of a supplement containing green‐lipped mussel (*Perna canaliculus*), curcumin (*Curcuma longa*) and blackcurrant (*Ribes nigrum*) leaf extract on locomotion and behaviour in client‐owned dogs and cats suffering from mild to moderate osteoarthritis. Overall, the supplement had some positive effects in reducing pain and lameness according to the owners’ observations in client‐owned dogs and cats with mild to moderate osteoarthritis.

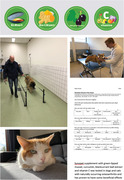

## 1 INTRODUCTION

Osteoarthritis is a common disease in dogs and cats. The prevalence of osteoarthritis in dogs is estimated to be about 20% in dogs under 1 year of age and 90% in dogs over 5 years of age, and in cats it is estimated to be 16.5%–91% in the total population, with increased prevalence with age (Johnson et al., 2020). These percentages seem to be stable over time, as a recent retrospective study evaluating the prevalence of osteoarthritis in cats in 1972–1973 showed similar results compared to more recent evaluations (Godfrey & Vaughan, 2018).

Osteoarthritis is a chronic progressive disease, which is treated by adaptation of the environment, weight reduction, exercise instructions, physical therapy and the use of non‐steroidal anti‐inflammatory drugs. As these drugs have side effects, especially with long‐term use, there is a need for alternative treatment options. Especially treatment options that focus on reducing the pathophysiological mechanisms of cartilage destruction, which also provide anabolic factors for cartilage repair, become more of interest. These treatment options may also be useful for prevention of osteoarthritis (Wang et al., 2018).

Glucosamine and chondroitin are the most commonly used supplements, with a recent systematic review demonstrating meaningful pain relief compared with control in humans (Ton et al., 2020). Suggested mechanisms of action of glucosamine and chondroitin are being building blocks of the cartilage glycosaminoglycan matrix, and reducing the activity of the cartilage degrading matrix metalloproteinases (MMPs). In green‐lipped mussel, glucosamine and chondroitin are combined with eicosatetraenoic acid (ETA) which is a long‐chain polyunsaturated omega‐3 fatty acid, and a precursor of eicosapentaenoic acid (EPA) and docosahexaenoic acid (DHA). These fatty acids are known for their anti‐inflammatory properties. Other components of green‐lipped mussel are furan fatty acids (F‐acids), sphingolipids, phytosterols, diacylglycerols, diterpenes, sesquiterpenes and saponins, alongside antioxidants such as carotenoids, xanthophylls and anthocyanins (Eason et al., 2018). Green‐lipped mussel was proven to be effective in dogs with naturally occurring osteoarthritis (Railland et al., 2013).

Curcumin has anti‐inflammatory properties and was proven to be effective in dogs with naturally occurring osteoarthritis (Innes et al., 2003). More recently, a study on 18 dogs with osteoarthritis has been performed. A phytosome complex form of curcumin was used, where a phospholipid was added to curcumin to enhance bioavailability. The outcome of this study showed a promising in vivo effect of curcumin. TNF‐α, NF‐kB1, IL‐8 and prostaglandin‐endoperoxide synthase 2 (PTGS2) all decreased in the curcumin‐treated group (Sgorlon et al., 2016).

Blackcurrant leaf extract contains several phytochemicals such as ProAnthoCyanidins (PACs) which dampen the inflammatory response and also have a protective effect on cartilage of people with osteoarthritis (Garbacki et al., 2002). These PACs also reduced adhesion molecules (ICAM‐1 and VCAM‐1), thus preventing immune cells to bind to the blood vessel wall (Garbacki et al., 2005).

A synergistic effect of phytochemicals and omega‐3 fatty acids has been suggested, as they all inhibit the inflammatory response and production of MMPs. The positive effects of these combinations on locomotion and pain in dogs and cats with osteoarthritis have been studied, both in therapeutic diets, as well as in supplements in both dogs and cats (Johnson et al., 2020). The combination of green‐lipped mussel, curcumin and blackcurrant leaf extract has to date not been studied in dogs and cats.

The aim of this study was to test the effect of a supplement containing green‐lipped mussel (*Perna canaliculus*), curcumin (*Curcuma longa*) and blackcurrant (*Ribes nigrum*) leaf extract on locomotion and behaviour in client‐owned dogs and cats suffering from mild to moderate osteoarthritis.

## 2 MATERIALS AND METHODS

Based on previous studies, we aimed at a minimum of 16 animals per group to finish the trials (Corbee et al. 2013; Musco et al. 2019). The animals were adults, had mild or moderate osteoarthritis at the start of the study and were otherwise healthy (based on physical exam performed by the author). Client‐owned dogs and cats were recruited via social media throughout the Netherlands and Belgium. Presence of mild to moderate osteoarthritis was based on clinical examination (physical examination and orthopaedic examination), as well as radiographic evidence of osteoarthritis which was required for inclusion in the study (i.e. a full Canine OsteoArthritis Staging Tool [COAST] score performed by the author). Dogs and cats with severe osteoarthritis and dogs weighing less than 10 kg were excluded. Dogs and cats that had used comparable supplementation or a mobility diet with similar ingredients in the past were excluded. Other diets or supplementation had to be stopped at least 2 weeks prior to the start of the study. The use of non‐steroidal anti‐inflammatory drugs (NSAIDs) was allowed, but only if they were used as rescue analgesia. Dogs and cats receiving permanent medication with NSAIDs were excluded.

The supplement (Synopet^®^ Cani‐Syn) contained the following ingredients per kilogram dry matter (93% water): 571,400 mg GLMax® Green‐lipped mussel (*Perna canaliculus*), 142,900 mg Bio‐CM100® Bio‐Curcumin (*Curcuma longa*), 238,571 mg Blackcurrant (*Ribes nigrum*) leaf extract, 242,857 mg vitamin C and 28,571 IU vitamin D3 (= 714 μg cholecalciferol). Liquid green‐lipped mussel was made in a cold process. Bio‐CM100^®^ Bio‐Curcumin consisted of more than 95% curcuminoids, plus turmeric essential oils to enhance its bioavailability (Antony et al. 2008). Citric acid, lactic acid and potassium sorbate were used as preservatives. Crude analysis: crude protein 2.3%, ether extract 0.3%, crude fibre <0.3%, crude ash 0.6% and moisture 93%. Placebo was made from the following ingredients: water, Locust bean gum, riboflavin (yellow colour), caramel (brown colour), chlorophyll (green colour) and synthetic fish aroma. Both the supplement and the placebo were provided by Synofit Europe B.V.

The effect of the supplement was tested in a double‐blinded, randomised, crossover, placebo‐controlled trial for 10 weeks per period in cats and 16 weeks per period in dogs. The animals first received either placebo or product, followed by a 2‐week wash‐out period, and then the other (similar to the protocol of Innes et al., 2003). Randomisation occurred according to the scheduled appointments. Both supplement and placebo had to be administered orally and in the same volume. The product and placebo were packed in identical packaging and were labelled with different lot numbers that were only known by the manufacturer and revealed after statistical analysis of the data. The liquid could either be mixed with the food or directly sprayed into the mouth of the dog using a syringe, as recommended by the manufacturer. The dosing schedule was based on weight and can be found in Table 1.

**TABLE 1:** Dosing schedule based on body weight

Weight	Days of treatment	Volume per day
<10 kg	25 days	3 ml
10–30 kg	40 days	5 ml
30–45 kg	40 days	7.5 ml
>45 kg	40 days	10 ml

Owners were required to keep a logbook to record each day whether the treatment was administered, if rescue analgesia were used and if there were any particularities.

All assessments and measurements were performed at the University Clinic for Companion Animals at Utrecht University at baseline, during the washout period and at the end of the study. All data collection was done by the same veterinarian assisted by a master student. Outcome measures were the Helsinki Chronic Pain Index (HCPI) in all dogs and cats (HCPI only study), the COAST and Force Plate Analysis (FPA) in dogs that were admitted to the clinic (full study).

### 2.1 Helsinki Chronic Pain Index

At each visit, owners were asked to fill in the HCPI score. This is a questionnaire consisting of 11 questions that relate to the mood, vocalisation of pain and several activities that are more difficult for dogs with chronic pain caused by osteoarthritis. An adapted HCPI score was used for cats (Corbee et al., 2013), where items were scored on a 5‐point scale (i.e. 1 = *much worse*, 2 = *worse*, 3 = *same*, 4 = *better*, 5 = *much better*) compared to baseline. To determine the stage of osteoarthritis at baseline, the cat owners were asked to give these scores compared to the period prior to osteoarthritis diagnosis. Both HCPI questionnaires are demonstrated in Appendix A. The dog HCPI questionnaire was adapted to giving a 1–10 score, as this is a common grading in the Netherlands. A score of 1 represents the worst condition, whereas a score of 10 represents the best condition. The final HCPI score of the items was divided by the number of items to get an overall average score between 1 and 10.

### 2.2 Canine OsteoArthritis Staging Tool

Clinical examination of dogs was used for staging the level of osteoarthritis by using COAST at each visit (Appendix B). Because there is quite a lot of variance of clinical symptoms within each stage (normal, mild, moderate and severe), half grades were added to allow for better distinction. A grade of 2.5, for example, would represent a dog with mild to moderate osteoarthritis. Because the supplement was not intended to affect the bony deterioration in osteoarthritis, radiography was not used in COAST staging of osteoarthritis during this study.

### 2.3 Force Plate Analysis

FPA was performed according to Corbee et al. (2014). The body weight of each dog was measured on a DIWAC VS150 electronic scale immediately before force plate measurement on every control moment. A quartz piezoelectric force plate (Kistler type 9261) with Kistler 9865B charge amplifiers, mounted flush in a walkway was used. The walkway was enclosed by a fence to guide the animal over the force plate. The standard platform measured 40 cm long and 60 cm wide. A firmly attached overlay plate with a length of 25 cm and a width of 60 cm was used for smaller dogs or dogs with a short stride length. The sampling rate was 100 Hz. Amplifiers were connected to a computer so that signals which corresponded with ground reaction forces in the vertical (Fz), craniocaudal (Fy) and mediolateral (Fx) directions could be recorded. Before data collection, equilibration and calibration of the force plate were performed according to the manufacturer's specifications. Dogs were guided by their owners over the force plate. They were instructed to walk faster or slower if necessary to perform good measurements. Measurement commencement and termination were automatically regulated by switches incorporated in the fence. Each pass across the platform that recorded either left or right forelimb, followed by the ipsilateral hindlimb, were saved. An observer confirmed if the right limbs had contacted the force plate and if contact of each foot was complete. Walking speed was between 1.0 and 1.2 m/s. Trials were discarded for incorrect walking speed, gait irregularities, partial loading of the plate or more than one foot striking the plate simultaneously. Trials were performed until 10 correct measurements of each limb were recorded. All ground reaction force data were normalised to body weight.

### 2.4 Statistical analysis

Statistical analysis was performed using SPSS statistics 25. All data were tested for normal distribution by a Kolmogorov–Smirnov test. Furthermore, data were looked at graphically using a distribution graph and compared to the standard normal distribution in a quantile–quantile plot. As forces are different between forelimb and hindlimb, the hindlimb data were corrected for fair comparison based on the percent difference in force as reported by Corbee et al. (2014) (i.e. 1.4 × Fzmax hindlimb = Fzmax forelimb). The Fzmax hindlimb was multiplied by 1.4 for fair comparison with Fz forelimb. HCPI, COAST and Fzmax of the worst affected limb were analysed by a linear mixed model. Dogs were indicated as subjects. The group effect was analysed by using group as a fixed factor. Data are presented as average and standard deviation, as well as the *p*‐value. Treatment effect was analysed by using treatment as a fixed factor, which was the best model fit. Differences between placebo and supplement compared to baseline are given. Item scores in cats were evaluated by a linear mixed model. Cats were indicated as subjects. The group effect was analysed by using group as a fixed factor. Data are presented as average and standard deviation, as well as the *p*‐value. Treatment effect was analysed by using treatment as a fixed factor, which was the best model fit. Differences between placebo and supplement are given, as there is no equal comparison possible with baseline data, because of the setup of the questionnaire. A *p*‐value of <0.05 was set as the level of significance, and a *p*‐value of ≤0.10 was considered a trend.

## 3 RESULTS

**FIGURE 1:**
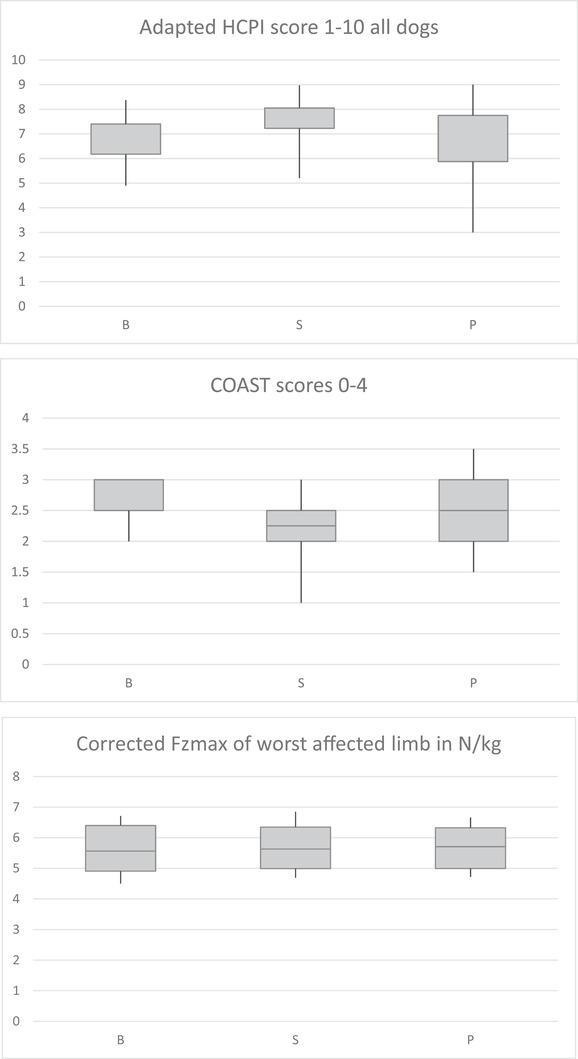
Adapted HCPI scores of all dogs, COAST scores and corrected Fzmax of the worst affected limb. Boxplots are demonstrated. HCPI, Helsinki Chronic Pain Index. This score has been adapted to grade 1–10 with 1 representing severe pain and 10 representing no obvious pain. This figure represents data of all 32 participating dogs. COAST, Canine OsteoArthritis Staging Tool: score 0 represents no signs of osteoarthritis and score 4 represents severe osteoarthritis. This figure represents data of 18 participating dogs. Fzmax is the peak vertical force measured by Force Plate Analysis and is expressed in N/kg. This figure represents data of 18 participating dogs. B, baseline; S, supplement; P, placebo

Of the 19 recruited dogs for the full study, 18 completed the study (Table 2). The dog that dropped‐out was diagnosed with cranial cruciate ligament rupture at the second control visit. Of the 20 dogs that were recruited for the HCPI only study, 14 completed the trial. The six dogs that dropped out were lost to follow‐up (owners did not respond to contact anymore). At baseline, a significant difference was present between groups for HCPI (7.95 ± 1.34 in the supplement‐placebo [SP] group compared to 6.69 ± 1.16 in the placebo‐supplement [PS] group, *p* < 0.001), which remained after treatment with either supplement (8.43 ± 1.29 and 7.52 ± 1.26, *p* < 0.001) or placebo (7.91 ± 2.18 and 6.73 ± 1.70, *p* < 0.001). The PS group had improved significantly greater on the supplement (0.83 ± 0.98) compared to the SP group (0.18 ± 1.73, *p* = 0.042), which was not the case for placebo treatment (0.04 ± 0.92 and –0.29 ± 1.55, *p* = 0.602). On COAST scores and FPA, there were no significant differences between PS and SP groups. The HCPI scores did not differ significantly in the dogs participating in the full study, but the COAST score improved significantly in the supplement group (*p* = 0.011), which was not the case in the placebo group (*p* = 0.103). The differences in COAST scores were not significantly different between supplement and placebo, as both demonstrated improvement (0.44 ± 0.17 and 0.28 ± 0.17, respectively, *p* = 0.324). No differences in FPA could be demonstrated (Table 3). The overall HCPI score in the 32 dogs (the 18 dogs of the full study combined with the 14 of the HCPI only study) improved with 0.67 ± 0.40 point in the supplement group, compared to 0.00 ± 0.40 in the placebo group (*p* = 0.100 and *p* = 0.994, respectively) (Table 4; Figure 1). Nine dogs did not show improvement in overall HCPI score (non‐responders), which was irrespective of their stage of osteoarthritis. None of the dogs received rescue NSAIDs during the trial.

**TABLE 2:** Description of the dog study population

	Breed	Age	Sex	Weight
1	Mixed breed	2	MN	17
2	Mixed breed	5	FN	24
3	Leonberger	10	FN	35
4	English Springer Spaniel	9	FN	19
5	Labrador Retriever	8	FN	34
6	Mixed breed	8	FN	34
7	Boxer	1	MN	29
8	Labradoodle	2	FN	20
9	Pitt Bull Terrier	2	FN	33
10	Welsh Corgi Pembroke	7	FN	16
11	Welsh Corgi Pembroke	1	FN	14
12	Golden Retriever	6	M	43
13	Belgian Shepherd	5	M	25
14	Labrador Retriever	1	M	34
15	Pug	10	M	11
16	Boxer	4	FN	27
17	Labrador Retriever	9	MN	30
18	Mixed breed	9	MN	30
19	Scottish shepherd	11	FN	20
20	Staffordshire terrier	2	F	14
21	Dutch shepherd	9	FN	37
22	Mixed breed	9	MN	28
23	American Bulldog	4	M	30
24	Rottweiler	6	FN	36
25	Malinois	5	M	32
26	Boxer	4	M	35
27	Labrador Retriever	6	MN	26
28	Bearded collie	11	FN	23
29	Sharpei	11	MN	22
30	Mixed breed	9	FN	25
31	Labrador Retriever	9	FN	25
32	Mastin Espagnol	2	F	47

*Note*: Individual dog data. Age in years, sex is Female (F), Female Neutered (FN), Male (M), Male Neutered (MN), weight in kg. Dogs 1–18 participated in the full study, dogs 19–32 participated in the HCPI only study.

**TABLE 3:** Results of the dogs from the full study

		HCPI			COAST			FPA Fzmax			
	Group	B	S	P	B	S	P	B	S	P	F/H
1	PS	8.2	9.4	7.8	3	3	3.5	5.32	4.99	5.18	H
2	SP	7.7	10	7.3	2.5	1	2	4.72	4.79	4.75	H
3	SP	8.1	8.9	8.8	3	2.5	3	6.58	6.55	6.37	F
4	PS	8.1	6.9	9.6	2	2	2.5	6.46	6.31	6.25	F
5	SP	9.7	9.2	9.3	3	2.5	3	5.11	4.99	4.97	H
6	PS	7.8	7.3	7.7	2.5	2.5	2	4.85	5.89	5.64	H
7	SP	8.6	9.8	8.4	3	3	3	5.42	5.57	4.82	H
8	SP	8.3	8	9.6	2.5	2	2	5.07	5.69	5.78	H
9	PS	7.2	8.7	6.9	3	2	2.5	5.71	5.17	5.8	H
10	SP	7.2	7.5	8	2	1.5	1.5	6.67	6.85	6.66	F
11	PS	7.6	8.3	7.3	2.5	1.5	1.5	6.22	6.36	6.35	F
12	PS	7.6	8.8	9.7	2	2	2	6.71	6.45	6.35	F
13	SP	9.7	10	10	2.5	2.5	2	6.69	6.64	6.43	F
14	SP	8.9	8.1	9	2.5	3	2.5	5.84	5.02	5.27	H
15	PS	5.2	7	5	3	2.5	3	5.81	6.01	6.23	F
16	SP	6.4	7.3	3.9	3	2	3	4.5	4.69	5.06	H
17	SP	8.6	9.4	9	2.5	2.5	2	4.78	4.81	4.72	H
18	PS	8.7	9.4	8.6	3	2	2.5	4.75	4.81	4.79	H
Average		7.98	8.56	8.11	2.64^a^	2.22^b^	2.42^a,b^	5.62	5.64	5.63	
SD		1.09	1.04	1.62	0.38	0.55	0.58	0.78	0.75	0.69	

*Note*: Individual dog data. Group is Placebo‐Supplement (PS) or Supplement‐Placebo (SP), respectively. B are baseline data, S is the difference between Baseline and Supplement and P is the difference between Baseline and Placebo. HCPI is the Helsinki Chronic Pain Index score, modified to get a score between 1 (severe pain) and 10 (no pain). COAST is the Canine OsteoArthritis Staging Tool with a score between 0 (no osteoarthritis) and 4 (severe osteoarthritis). FPA Fzmax is the maximum peak vertical force (average of the front limbs) on Force Plate Analysis expressed in N/kg. Differences between ^a^ and ^b^ were considered significant (*p* = 0.011), whereas groups with similar letters did not differ.

**TABLE 4:** Results of the dogs from the HCPI only study, and combined with the HCPI results from Table 3

	Group	B	S	P
19	PS	6.7	7.3	7.9
20	SP	7	7.6	7.8
21	PS	5.8	5.3	5.8
22	SP	5.7	5.2	3
23	PS	5.3	6.2	5.6
24	PS	6.1	8.9	6.5
25	SP	8.9	8.1	9.9
26	PS	6.5	7.4	6
27	PS	5.7	6.5	3.7
28	PS	4.9	6.1	4.3
29	SP	5.5	8	5.2
30	SP	9	9.4	9.4
31	SP	6.2	6.2	6.1
32	PS	6.2	8.2	5.9
Average		6.39	7.17	6.22
SD		1.21	1.29	1.99
Overall				
Average		7.28^a^	7.95^b,x^	7.28^y^
SD		1.38	1.34	2.00

*Note*: Individual dog data. Group is Placebo‐Supplement (PS) or Supplement‐Placebo (SP), respectively. B are baseline data, S is the difference between Baseline and Supplement and P is the difference between Baseline and Placebo. HCPI is the Helsinki Chronic Pain Index score, modified to get a score between 1 (severe pain) and 10 (no pain). Differences between ^a^ and ^b^ were considered a trend (*p* = 0.100). Differences between x and y were considered a trend (*p* = 0.098).

Of the 26 recruited cats, 16 completed the study (Table 5). Of the 10 cats that dropped‐out, six owners were unable to administer the supplement due to palatability or other issues, 2 cats died of non‐osteoarthritis related causes, and 2 were lost to follow‐up as the owners did not show up on follow‐up visits. In cats, improvement of some of the HCPI parameters were demonstrated in the supplement group compared to the placebo group, i.e. the ability to groom (3.64 ± 0.63 vs 3.00 ± 0.38, *p* = 0.009), activity level (3.73 ± 0.80 vs 3.19 ± 0.66, *p* = 0.017), playfulness (3.67 ± 0.82 vs 2.92 ± 0.28, *p* = 0.002), walking up the stairs (3.43 ± 0.65 vs 3.00 ± 0.38, *p* = 0.039), and overall level of satisfaction (3.53 ± 0.65 vs 3.06 ± 0.68, *p* = 0.025). Four cats did not show improvement on any of the parameters, which was irrespective of their stage of osteoarthritis. None of the cats received rescue NSAIDs during the trial.

**TABLE 5:** Description of the cat study population

	Breed	Age	Sex	Weight
1	Turkish Van	10	MN	6.2
2	DSH	15	MN	4.7
3	Maine Coon	4	MN	10
4	DSH	15	FN	3.9
5	DSH	17	FN	4.2
6	Turkish Van	8	MN	6.1
7	DSH	13	FN	6
8	DSH	17	MN	4.7
9	Maine Coon	15	FN	5.3
10	DSH	16	MN	5.6
11	Maine Coon	9	MN	10.3
12	DSH	15	MN	5.4
13	DSH	8	MN	6.5
14	DSH	12	MN	5.8
15	Birman	17	FN	6.5
16	DSH	15	MN	5.2

*Note*: Individual cat data. DSH is Domestic Shorthair, age in years, sex is Female Neutered (FN), Male Neutered (MN), weight in kg.

## 4 DISCUSSION

The aim of this study was to test the effect of a supplement containing green‐lipped mussel (*Perna canaliculus*), curcumin (*Curcuma longa*) and blackcurrant (*Ribes nigrum*) leaf extract on locomotion and behaviour in client‐owned dogs and cats suffering from mild to moderate osteoarthritis. Based on the trend for improvement of the overall HCPI score in dogs, and improvement on several HCPI items in cats, the owners noted clinical improvement. COAST scores improved when using the supplement, but there was also improvement in the placebo group resulting in no significant difference between supplement and placebo. Furthermore, FPA did not show significant changes. Non‐responders were present in both dogs and cats irrespective of the stage of osteoarthritis. The results of non‐responders should not be ignored or censored but should be taken into account when interpreting the overall effect, as non‐responders have a negative impact on the overall observed effect of the supplement. In some individuals, improvement was also demonstrated with placebo. This can be explained by the caregiver placebo effect (Conzemius & Evans 2012, Gruen et al., 2017). This effect narrows the difference between the supplement group and the placebo group, which also stresses the importance of comparison with baseline.

Several nutraceuticals have been proven to be effective in dogs, of which omega‐3 fatty acids are considered among the most promising (Johnson et al., 2020).

Little research has been done on the effects of nutraceuticals in cats with osteoarthritis. The results of this study were similar to the findings in cats that were given omega‐3 fatty acids, as with both supplements cats revealed a higher activity level, and more walking up the stairs. Effects that were demonstrated with the supplement in this study and not with omega‐3 supplementation were increased ability to groom, playfulness and overall level of satisfaction, whereas with omega‐3 supplementation less stiffness during gait, more interaction with the owner and higher jumps were noted, which were not seen with the supplement in this study (Corbee et al., 2013). Comparison with baseline was not possible due to the setup of the questionnaire, which was a limitation of the study.

Because of the high variation between individual responses, a significant difference could not be found in HCPI and FPA. For HCPI, a significant difference might be expected to be found with a larger sample size, as the HCPI results of the full study already demonstrated a trend. It could be noted that COAST is designed as a staging tool, and maybe more useful for selecting the patients for the study rather than evaluating the effect of nutraceuticals or other therapeutic agents. Whether the observed average 0.44‐point improvement of the COAST score is clinically relevant therefore remains speculative. FPA results did not show significant differences between groups, nor between supplement, placebo and baseline. FPA is complex and its results are dependent on the stage of osteoarthritis, the location of the osteoarthritis and the compensation by other limbs. The peak vertical force is mostly used as outcome parameter; however, this parameter is also influenced by several factors, such as symmetry, division of the weight bearing over the four limbs, the impulse and walking speed.

Only a single dosage of the supplement was tested. It is possible that a higher dosage would have given a better result. Also, the duration of the study was quite short, as usually supplements for osteoarthritis in dogs and cats are evaluated after a 3‐ to 6‐month period.

Further research with a larger sample size, different dosage and longer duration is therefore needed to expand the findings from this study.

In conclusion, the supplement had some positive effects but minimal significant findings in reducing pain and lameness according to the owners’ observations in client‐owned dogs and cats with mild to moderate osteoarthritis.

## ETHICAL STATEMENT

The study was approved according to Dutch legislation and registered under number AVD1080020184847.

## AUTHOR CONTRIBUTION

Dr. R.J. Corbee was responsible for conceptualization, formal analysis, funding acquisition, investigation, methodology, project administration and writing.

### PEER REVIEW

The peer review history for this article is available at https://publons.com/publon/10.1002/vms3.779.

## Data Availability

The data that support the findings of this study are available from the corresponding author upon reasonable request.

## APPENDIX A

### Helsinki Chronic Pain Index

**Table:**

Name owner:
Name dog:
Date:
Control moment: 1 / 2 / 3

**Table:**

Question	10	Gradation		1		Points
Rate your dog's mood.	Very alert	Alert	Neutral	Indifferent	Very indifferent	
Rate your dog's willingness to participate in play.	Very willing	Willing	Reluctantly	Very reluctantly	Does not play at all	
How often does your dog groan, squeal or whine?	Never	Hardly ever	Sometimes	Often	Very often	
Rate your dog's willingness to walk.	Very willing	Willingly	Reluctantly	Very reluctantly	Does not walk at all	
Rate your dog's willingness/ability to walk up and down stairs.	Very willing	Willingly	Reluctantly	Very reluctantly	Does not want to at all	
Rate your dog's willingness to run.	Very willing	Willingly	Reluctantly	Very reluctantly	Does not run at all	
Rate your dog's willingness to jump (e.g. into the car or onto the sofa).	Very willing	Willingly	Reluctantly	Very reluctantly	Does not jump at all	
Rate your dog's ease in lying down.	With great ease	Easily	Neutral	With difficulty	With great difficulty	
Rate your dog's ease in rising from a lying position.	With great ease	Easily	Neutral	With difficulty	With great difficulty	
How difficult is it for your dog to move after a long period of rest?	Never difficult	Hardly ever difficult	Sometimes difficult	Often difficult	Very often or always difficult	
How difficult is it for your dog to move after major activity or heavy exercise?	Never difficult	Hardly ever difficult	Sometimes difficult	Often difficult	Very often or always difficult	
					Total	

### Cat questionnaire

1. Does your cat live indoors, outdoors or both?
2. How long is your cat outdoors: 0 hour, 0–3 hours, more than 3 hours?
3. Did you notice changes with allowing you to groom the cat?
4. Does your cat sometimes defecate outside the edge of the litter box? (yes/no)
5. Did you notice any changes with that?
6. Did you notice a difference in playing with other pets/people?
7. Did you notice a difference in activity level of the cat?
8. Did you notice any changes in jumping on table, couch, windowsill etc.?
9. Does your cat jump from a lower height? (yes/no)
10. Did you notice any changes in the height of jumps?
11. Did you notice any changes in accessibility of the cat's favourite spots within the house?
12. Did you notice any changes in walking up the stairs?
13. Did you notice any changes in walking down the stairs?
14. Did you notice any changes in stiffness of the cat's gait?
15. Did you notice any changes in aggressive behaviour?
16. Did you notice any changes in satisfaction level of your cat?
17. Did you notice any changes in greeting people?
18. Did you notice any changes in allowing to be petted by people?
19. Did you notice any changes in interaction with other cats?
20. Did you notice changes in the ability to groom itself?
21. Did you notice changes in the time spend that the cat is grooming itself?
22. Has your cat ever been hit by a car? (yes/no)
23. Has your cat been lame in the past? (yes/no)
24. Is your cat lame at the moment? (yes/no)

Questions 1, 2, 22, 23 and 24 were asked only once.

Answers were categorised in: more, a bit more, equal, a bit less, less

Answers to questions 5, 14 and 15 were inverted prior to analysis

## APPENDIX B

### Canine OsteoArthritis Staging Tool (COAST)

**Table:**

‘Grade the dog’						Grade
I	Effect on posture (static)	Normal; Static posture appropriate for breed Appropriate limb loading Appropriate body weight distribution between forelimbs and hindlimbs	Mildly abnormal; Subtle abnormality of limb loading Subtle shift in static body weight distribution	Moderately abnormal; Obvious abnormality in limb loading Obvious shift in static body weight distribution	Severely abnormal; Restless when standing Reluctance (difficulty) to stay standing Severe shift in static body weight distribution Severely abnormal limb loading	(1–4)
II	Effect on motion	Normal; Symmetry Appropriate weight bearing Appropriate body weight distribution Fluent gait	Mildly abnormal; Motion possibly affected at some gaits or with some activities Subtle stiffness in gait Subtle changes in body weight distribution Subtle asymmetry Subtle lameness No difficulty rising (getting up)	Moderately abnormal; Consistent abnormalities in motion at all gaits and activities Obvious stiffness in gait Obvious changes in body weight distribution Obvious reduction in use of affected limb Obvious decrease in stance phase Some difficulty rising (getting up)	Severely abnormal; Struggles to move/reluctant to move Severe lameness usually present Severe weight shift Marked difficulty rising (getting up)	(1–4)

‘Grade the joint’						Grade
III	Pain upon manipulation	None	Mild	Moderate	Severe	(1–4)
IV	Passive range of motion	Normal	Mildly abnormal; Minimally reduced ROM No crepitus Slight joint thickening	Moderately abnormal; Obvious decrease in ROM Muscle atrophy Obvious joint thickening	Severely abnormal; Extremely limited ROM Crepitus Extreme muscle atrophy Severe joint thickening Loss of anatomical normality upon palpation Anatomical misalignment	(1–4)
V	Radiography	No radiographic signs of OA; If preclinical ‘at risk’, the dog may have radiographic evidence of risk factors such as dysplasia and/or trauma	Mildly abnormal; Early signs of OA Minimal osteophytes	Moderately abnormal; Obvious osteophytes	Severely abnormal; Advanced osteophytes Remodelling	(1–4)

**Table:**

Stage of OA	Description	
0	Preclinical	No risk factors apparent
1		‘At risk’: At least one predisposing factor for OA apparent, e.g. breed predisposition, joint injury, obesity, intense activity and/or radiographic signs of dysplasia or joint trauma
2	Mild	
3	Moderate	
4	Severe	
